# Manual therapy and exercise effects on inflammatory cytokines: a narrative overview

**DOI:** 10.3389/fresc.2024.1305925

**Published:** 2024-04-30

**Authors:** Chad E. Cook, Damian Keter, William Todd Cade, Beth A. Winkelstein, William R. Reed

**Affiliations:** ^1^ Doctor of Physical Therapy Division, Department of Orthopaedics, Duke University, Durham, NC, United States; ^2^ Department of Population Health Sciences, Duke University, Durham, NC, United States; ^3^ Duke Clinical Research Institute, Duke University, Durham, NC, United States; ^4^ Department of Veterans Affairs Medical Center, Cleveland, OH, United States; ^5^ Departments of Bioengineering & Neurosurgery, University of Pennsylvania, Philadelphia, PA, United States; ^6^ Department of Physical Therapy, University of Alabama at Birmingham, Birmingham, AL, United States

**Keywords:** precision medicine, pain, cytokines, manual therapy, exercise, musculoskeletal

## Abstract

**Background:**

Matching disease and treatment mechanisms is a goal of the Precision Medicine Initiative. Pro- and anti-inflammatory cytokines (e.g., Tumor Necrosis Factor-alpha, Transforming Growth Factor-beta, and Interleukin-2, 10, and 12) have gained a significant amount of interest in their potential role in persistent pain for musculoskeletal (MSK) conditions. Manual therapy (MT) and exercise are two guideline-recommended approaches for treating MSK conditions. The objective of this narrative overview was to investigate of the effects of MT and exercise on pro- and anti-inflammatory cytokines and determine the factors that lead to variability in results.

**Methods:**

Two reviewers evaluated the direction and variabilities of MT and exercise literature. A red, yellow, and green light scoring system was used to define consistencies.

**Results:**

Consistencies in responses were seen with acute and chronic exercise and both pro- and anti-inflammatory cytokines. Chronic exercise is associated with a consistent shift towards a more anti-inflammatory cytokine profile (Transforming Growth Factor-beta, and Interleukin-2 and 13, whereas acute bouts of intense exercise can transiently increase pro-inflammatory cytokine levels. The influence of MT on cytokines was less commonly studied and yielded more variable results.

**Conclusion:**

Variability in findings is likely related to the subject and their baseline condition or disease, when measurement occurs, and the exercise intensity, duration, and an individual's overall health and fitness.

## Introduction

The Precision Medicine Initiative is an emerging approach for disease prevention and treatment that considers individual differences in people's genetic background, environments, and lifestyles (1). To date, most precision medicine-based approaches for musculoskeletal-related (MSK-related) pain disorders remain in the early stages of development and implementation. Nonetheless, recent successes (2–4) have elevated interests and have highlighted the importance of matching predictive biomarkers such as pro- and anti-inflammatory cytokines, especially for matching MSK-related conditions with dedicated treatment mechanisms (5–7). Conceptually, by exploring pro- and anti-inflammatory cytokines in patients, we may be able to tailor treatments and dosages, thus improving post-treatment responses (8–10).

Interventions such as manual therapy (MT) (e.g., manipulation, mobilization and massage) and exercise (e.g., resistance and aerobic) are typically recommended by clinical guidelines, as first-line approaches for management of MSK-related pain (11, 12). Both approaches have theoretically different mechanisms by which they provide clinical efficacy. Manual therapy activates both peripheral and central physiological mechanisms, which have shown to reduce pain, muscle hypertonicity, and improve mobility (13). Exercise occurs in many forms but is commonly labeled as resistance exercise or aerobic. Resistance exercise leads to increases in muscle fiber size and neural adaptations, thus improving strength and endurance (13). Aerobic exercise releases endorphins, improves blood flow, and reduces hypoxia and macrophage infiltration (14).

Cytokines are a large family of small-secreted proteins produced by numerous cells that function independently and interact with immune-related cells. Other terms within the cytokine's family include lymphokines, monokines, chemokines, and interleukins, which reflect the cells from which they are generated (8, 15). Cytokines are essential in regulating the human immune response (15) and are broadly categorized as pro-inflammatory (Table 1) or anti-inflammatory (Table 2) (15–17).

**Table 1:** Pro-inflammatory cytokines in humans.

Name	Role
Tumor necrosis factor-alpha (TNF-α)	TNF-α is produced by various immune cells, including macrophages and *T* cells. It plays a crucial role in initiating inflammation and is associated with many inflammatory diseases, as well as pain.
Interferon-gamma (IFN-γ)	IFN-γ plays a crucial role in regulating the immune response, particularly in coordinating the activities of immune cells, such as macrophages and *T* cells, to combat infections and control inflammation.
Interleukin-1 (IL-1α or IL-1β)	Macrophages and other immune cells produce IL-1. It stimulates inflammation by promoting the activation of immune cells, inducing fever, and promoting tissue repair.
Interleukin-6 (IL-6)	Multiple cell types produce IL-6, including immune cells and certain non-immune cells. It plays a role in promoting inflammation, regulating the immune response, and influencing the production of acute-phase proteins.
Interleukin-8 (IL-8)	Immune cells primarily produce IL-8, which acts as a chemoattractant, recruiting other immune cells to sites of inflammation and infection.
Interleukin-12 (IL-12)	IL-12 is produced by antigen-presenting cells such as dendritic cells and macrophages. It promotes the differentiation of T cells into a pro-inflammatory type (Th1), which helps enhance the immune response against intracellular pathogens.
Interleukin-17 (IL-17)	IL-17 is produced by a subset of immune cells known as T helper 17 (Th17) cells, as well as other cell types such as natural killer T cells, and innate lymphoid cells. IL-17 promotes inflammation and recruits immune cells to sites of infection or tissue damage.
Interleukin-18 (IL-18)	IL-18 plays a significant role in regulating the immune response, particularly in the context of inflammation, infection, and immune cell communication.
Interleukin-22 (IL-22)	IL-22 is a cytokine, which is a type of signaling molecule that plays a crucial role in the immune system. It is part of the IL-10 family of cytokines and is produced by various immune cells, including *T* cells and natural killer (NK) cells.
Interleukin-23 (IL-23)	IL-23 belongs to the interleukin-12 (IL-12) family of cytokines, is produced by antigen-presenting cells, such as dendritic cells and macrophages, and interacts with specific immune cells to modulate immune reactions.
Granulocyte-macrophage colony-stimulating factor	GM-CSF plays a crucial role in the regulation and stimulation of the production, differentiation, and function of various immune cells, particularly granulocytes and macrophages.

**Table 2:** Anti-Inflammatory cytokines in humans.

Type name	Role
Interleukin-2 (IL-2)	IL-2 is a cytokine that plays a crucial role in regulating the immune system. It is produced by certain immune cells, primarily T cells, which are a type of white blood cell involved in immune responses.
Interleukin-4 (IL-4)	Although IL-4 is often associated with promoting Th2-type immune responses, it also has anti-inflammatory properties. It can suppress the production of pro-inflammatory cytokines and promote the development of regulatory immune cells.
Interleukin-10 (IL-10)	Although it is often considered an anti-inflammatory cytokine, IL-10 also has complex roles in regulating inflammation. It helps control excessive inflammation and can have both pro- and anti-inflammatory effects depending on the context.
Interleukin-13 (IL-13)	IL-13 is part of the interleukin-4 (IL-4) cytokine family. It plays a significant role in regulating immune responses, particularly those related to allergic and anti-parasitic responses, as well as tissue repair and inflammation.
Transforming growth factor-beta (TGF-β)	TGF-β has both anti-inflammatory and pro-inflammatory effects, depending on the context. It plays a role in wound healing and tissue repair.

Pro- and anti-inflammatory cytokines have contrasting effects on inflammation and immune reactions, and over different temporal phases. Pro-inflammatory cytokines promote inflammation, aimed at eliminating harmful agents and damaged cells, as well as initiating tissue repair. Pro-inflammatory cytokines also directly or indirectly sensitize nociceptors both peripherally and centrally (18) and may play a role in depression, anxiety, sleep disturbances (19) and cognitive deficits (18). Whereas inflammation serves a protective function initially, excessive or prolonged inflammation can lead to tissue damage and contribute to various chronic diseases such as autoimmune disorders, allergies, neuronal dysfunction, psychiatric disorders and certain types of cancers (8, 15). Anti-inflammatory cytokines (e.g., Transforming Growth Factor-beta, Interleukin-4, 10 and 13) act as counterbalances to pro-inflammatory cytokines (e.g., Tumor Necrosis Factor-alpha, Interleukin-1, 6, 8 and 12), helping to prevent excessive inflammation and promoting immune system homeostasis (8). Some cytokines can act as either pro- or anti-inflammatory, depending on the physiological circumstance or both in selected situations (8).

Cytokines are known to play a role in MSK-related pain (5–7). Low-grade inflammation is associated with the severity of low back and neck pain (18, 20–23) and specific conditions such as intervertebral disc degeneration has a complex inflammatory response within its pathological processes, with elevated levels of pro-inflammatory cytokines (24). Several pro-inflammatory mediators have been identified as playing a role in rotator cuff tendinopathy (25) and general shoulder pain (26). Dominate nociplastic pain conditions such as fibromyalgia exhibit different circulating cytokine profiles versus healthy controls (27). Conflicting evidence exists regarding the role of inflammatory cytokines in knee osteoarthritis (28).

Prescription drugs are used to target specific pro- and anti-inflammatory cytokines. Adalimumab (brand names Humira, Amgevita, Hyrimoz, Idacio, Imraldi, and Yuflyma), which is used to treat conditions such as Crohn's disease and rheumatoid arthritis, targets and inhibits tumor necrosis factor-alpha (TNF-α). Tocilizumab (brand name Actemra), which is used to treat rheumatoid and systemic juvenile arthritis, targets interleukin-6 (IL-6) receptors. Prednisolone is used in treatment of a number of inflammatory conditions and can suppress the production of pro-inflammatory cytokines such as interleukin-1 (IL-1), interleukin-6 (IL-6), and tumor necrosis factor-alpha (TNF-α). Anti-TNF-α therapy agents are used in alleviating intervertebral disc degeneration, especially in inhibiting extracellular matrix degradation and reducing inflammatory responses (24).

Laboratory and preclinical studies have shown that MT and exercise can modulate pro- and anti-inflammatory cytokines as well (29–38); however, the extent of the modulation and the direction of influence (i.e., increase or decrease in cytokine levels) is largely unknown. The purpose of this narrative overview is to investigate of the effects of MT and exercise on pro- and anti-inflammatory cytokines. We reviewed and summarized the current pre-clinical and clinical evidence evaluating MT and exercise influences on cytokines and included studies that outlined the direction of the influence as well as factors that might explain why differences were present in the literature.

## Materials and methods

### Design

The study used a narrative overview design. Narrative overviews are useful as they compile large amounts of information together into a structured, transferable format that includes identification of gaps in knowledge and present trends (39–41). Narrative overviews are especially useful for (1) developing theories of how an intervention works, why and for whom; (2) developing a preliminary synthesis of findings of included studies; or (3) exploring relationships in the data (42). Unlike systematic reviews, narrative overviews rarely include a risk of bias score and routinely do not involve an automated systematic search (39).

### Search strategy

We included articles published in PubMed, and those referenced in systematic reviews. Systematic reviews were also included, if they reported the direction of the influence of MT or exercise on cytokines. A biomedical librarian was consulted in August of 2023 to assist in the structure of the search. The search string included keywords (“inflammatory cytokines” OR “pro-inflammatory cytokines” OR “anti-inflammatory cytokines”) AND (exercise OR “physical activity” OR “exercise therapy” OR “exercise training”) AND (manual therapy OR “massage therapy” OR “manipulative therapy” OR “chiropractic therapy”).

### Study inclusion criteria

We included studies that incorporated short-term or longer-term applications of MT and/or exercise (acute and chronic) and measured cytokine changes after application. All experimental designs were considered, including both pre-clinical and clinical research, with any follow-up period. We included all approaches used in collection and measurement of inflammatory cytokines [e.g., enzyme-linked immunosorbent assay (ELISA), urine, etc.] (43).

### Subject inclusion criteria

We included both animal and human studies, and did not restrict papers to those involving MSK-related disorders, since there are very few in the literature. Since one of the goals of this narrative was to determine the influence of MT and exercise on cytokines, we included studies with animal models and humans with metabolic disorders, of all ages, healthy controls, and individuals who had known inflammatory conditions such as fibromyalgia.

### Selection of specific pro- and anti-inflammatory cytokines

We limited pro- and anti-inflammatory cytokines to those routinely captured on an Enzyme-linked immunosorbent assay (ELISA) that are thought to have high relevance in various biological processes and diseases (Tables 1, 2).

### Selection of specific MT and exercise interventions

We included all forms of acute and chronic (regular) MT and exercise and did not restrict studies based on parameters of intervention application. We did not evaluate the fidelity of the MT and/or exercise interventions.

### Specific measures-direction of influence

Direction of influence (e.g., how MT and exercise influenced changes in cytokines) was recorded using a novel green, yellow and red-light assessment, independently by two authors (CC, DK). A score of “green light” represented one or more studies that (consistently) showed a defined pattern of either decreasing or increasing plasma cytokine levels after application of MT or exercise. A score of “red light” suggested there was one or more studies that did not find any change (either from baseline or against a comparator) in cytokines after application of MT or exercise (in other words, MT and exercise did not influence the cytokine). A score of “yellow light” either consisted of “mixed” or conflicting information (e.g., involving an increase, decrease, or no-response) across two or more studies. Studies could have a green, yellow or red light and a designation of “limited” evidence, if there are few studies that have explored the role of MT and exercise on the cytokine of interest.

### Specific measures-factors that identified variations in findings

Two authors (CC, DK) used a modified form of thematic coding to explain factors that might explain why differences were present in the literature. In particular, we evaluated potential differences in: (1) follow up timing, (2) subject health, (3) types of MT and exercise applications used, including force, intensity, etc., (4) how long the interventions were provided, and (5) how the cytokines were measured.

## Results

### Study inclusion

Our search identified 4,434 studies involving exercise and cytokines (128 systematic reviews) and 2,437 studies involving MT and cytokines (28 systematic reviews). Variability across studies was very high; studies involved preclinical and clinical research, animal and human models, and a wide range of MT and exercise applications. To summarize the evidence in this overview, we included comparative trials, pre-post studies, and systematic reviews.

### Evidence for pro-inflammatory cytokine modulation with manual therapy and exercise

Table 3 outlines the current evidence for MT and exercise for modulation of pro-inflammatory cytokines (29–54, 56–82, 91–110). A majority of studies found that acute (short-term) exercise may lead to transient changes in both pro- and anti-inflammatory cytokine levels, whereas chronic (regular) exercise of various forms, especially at moderate intensities, is generally associated with a reduction in pro-inflammatory cytokine profiles. There is mixed evidence that TNF-a, IFN-y, IL-6, and IL-8 are influenced by MT approaches. The direction of the influence is conflicting. Outside of IL-18 and IL-22, which have presently not been studied, the remaining pro-inflammatory markers do not appear to be influenced by MT—although there are a limited number of studies that have investigated such relationships.

**Table 3:** Evidence suggesting an association between exercise, manual therapy and Pro-inflammatory cytokines.

Cytokine	Exercise		Manual therapy	Strength of evidence
	Effect and references	Strength of evidence	Effect and references	
Tumor necrosis factor-alpha (TNF-α):	Mixed evidence, increased during short-term, intense exercise, decreased during regular exercise training, when performed consistently over time. Is highly influenced by exercise type and by comorbidities (37, 44–55).	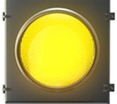	Mixed evidence across multiple studies that manual therapy influences TNF-α in the short term. Results vary depending on the technique selected and the comorbidities of the patient (29, 31, 33, 56–59).	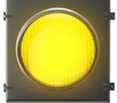
Interferon-gamma (IFN-γ)	Limited evidence but findings suggest a reduction of IFN-γ after intense exercise (55).	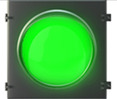	Mixed evidence between how manual therapy influences IFN-γ levels after intervention (29, 32, 61, 62).	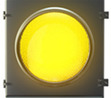
Interleukin-1 (IL-1α or IL-1β):	Mixed, but mostly increased during short-term, intense exercise, decreased during regular exercise training, when performed consistently over time (37, 45, 46, 49, 50, 55, 63–65).	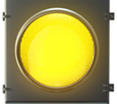	Mixed evidence suggesting a potential link between manual therapy and modulation of IL-1β (29, 31, 56–59, 62).	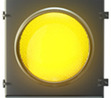
Interleukin-6 (IL-6):	Mixed but mostly increased during short-term, intense exercise, no effect or stimulation of IL-6 during regular, consistent exercise, over time (46, 50–52, 56, 62, 66–81).	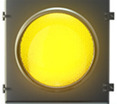	Mixed evidence suggesting manual therapy effects IL-6 up to 20 min after intervention (29, 33, 57, 58, 82).	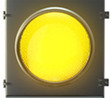
Interleukin-8 (IL-8):	Mixed but mostly increased during short-term, intense exercise, and decreased during regular exercise training, when performed consistently over time. Is highly mediated by exercise type and by comorbidities (63).	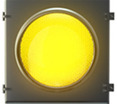	The two studies that have investigated the modulating role of manual therapy on IL-8 show no effect (27, 29).	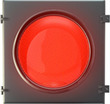
Interleukin-12 (IL-12):	Increased during short-term, intense exercise, and decreased during regular exercise training, when performed consistently over time (63).	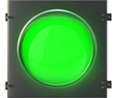	To date, one study has shown nominal effect of manual therapy on IL-12 (29).	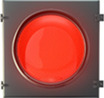
Interleukin 17 (IL-17)	Increased during short-term, intense exercise, and decreased during regular exercise training, when performed consistently over time (83–85).	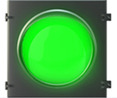	There are currently no studies that support that manual therapy modulates IL-17 (29, 32).	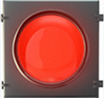
Interleukin 18 (IL-18)	Increased during short-term, intense exercise, and decreased during regular exercise training, when performed consistently over time (55, 86, 87).	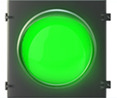	We were unable to find any studies that have investigated whether manual therapy affects IL-18.	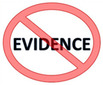
Interleukin 22 (IL-22)	Increased during short-term exercise, and decreased during regular exercise training, when performed consistently over time (88, 89).	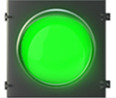	We were unable to find any studies that have investigated whether manual therapy affects IL-22.	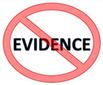
Interleukin 23 (IL-23)	Temporality increased during short-term exercise, and decreased during regular exercise training, when performed consistently over time (88).	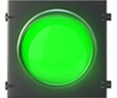	To date, one study has shown nominal effect of manual therapy on IL-23 (29).	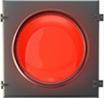
Granulocyte-macrophage colony stimulating factor (GM-CSF)	Mixed, but mostly temporality increased during short-term exercise, and decreased during regular exercise training, when performed consistently over time (51, 90).	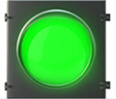	To date, one study has shown nominal effect of manual therapy on GM-CSF (29).	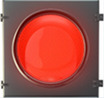

### Evidence for anti-inflammatory cytokine modulation with manual therapy and exercise

Table 4 outlines the current evidence for MT and exercise for modulation of anti-inflammatory cytokines (29–31, 33, 44, 47, 49–51, 53, 55, 58, 61–63, 66, 67, 77, 79, 80, 91, 101, 111–142). Current evidence suggests acute exercise tends to increase anti-inflammatory cytokines (i.e., IL-10, IL-13, and TGF-β). Studies suggest that IL-2 and IL-4 increase with MT, whereas values are mixed or unstudied in other cytokines.

**Table 4:** Evidence suggesting an association between exercise, manual therapy and anti-inflammatory cytokines.

Cytokine	Exercise		Manual therapy	Strength of evidence
	Effect and references	Strength of evidence	Effect and references	
Interleukin-2 (IL-2)	Limited evidence suggesting a reduction in IL-2 with exercise; mostly studied in aerobic exercise (55).	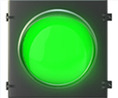	All studies show manual therapy increases levels of IL-2 after intervention (33, 62, 66, 91).	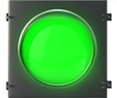
Interleukin-4 (IL-4):	Mixed evidence of changes with short- or long-term exercises on IL-4 (55).	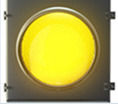	Current evidence suggests that manual therapy decreases IL-4 after intervention (62).	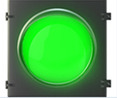
Interleukin-10 (IL-10)	Mixed evidence on how exercise influences IL-10 levels after application, which is dependent on type and intensity. Most studies show an increase in levels (44, 47, 49–51, 53, 55, 63, 67, 77, 79, 80, 101).	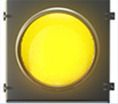	Mixed evidence between how manual therapy influences IL-10 levels after intervention (29–31, 58, 61).	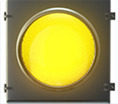
Interleukin-13 (IL-13)	A majority of research suggests IL-13 is increased with both acute and long-term exercise; however, population-based variation is demonstrated (111–121).	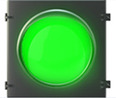	Mixed evidence between how manual therapy influences IL-13 levels after intervention (29, 62).	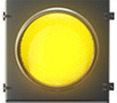
Transforming growth factor-beta (TGF-β)	A majority of research suggests TGF-β is increased with both acute and long-term exercise and differs based on exercise intensity; however, population-based variation is demonstrated (122–142).	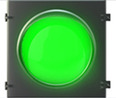	We found no evidence investigating the effect of manual therapy of TGF-β (110).	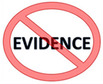

### Factors that identified variations in findings

For exercise and pro-inflammatory cytokines, the amount of change depends on various factors, including the subject and their baseline condition or disease, when measurement occurs, and the exercise intensity, duration, and an individual’s overall health and fitness. The variations in findings for pro-inflammatory cytokines and MT may be explained by differences in techniques used, differences in intensities and variations in subject populations, including baseline condition, context of the MT provided, when measurement occurs, and follow-up times. For anti-inflammatory cytokines, similar types of variations present in pro-inflammatory literature were likely the reason for mixed results in selected cases.

## Discussion

The purpose of the study was to investigate the effects of MT and exercise on pro- and anti-inflammatory cytokines. We were particularly interested in measuring direction of the influence as well as factors that might explain why differences were present in the literature. As anticipated, we found notably more studies investigating the role of exercise on cytokine levels/profile, as compared to MT approaches indicating the need for additional investigation. The experimental design types were highly variant, involving both pre-clinical and clinical studies, with study participants with and without health-related conditions.

### Exercise and MT and their influence on cytokines

There was a notable trend toward an increase in pro-inflammatory cytokine levels with acute exercise, and a reduction in cytokine levels with longer-term, chronic exercise (51, 55, 60, 63, 83–90). A consistent pattern involving increases emerged for anti-inflammatory cytokines with acute and regular aerobic and resistance exercise (111–142). Generally, moderate-intensity aerobic exercise and resistance training is associated with anti-inflammatory effects; however, excessive or intense exercise without adequate recovery appeared to increase inflammation over the short term. This suggests that exercise may be useful as a moderator for inflammation that occurs as a result of infections, injuries, or stress, but dosing (intensity and timing) is critical.

The most accurate way to summarize the role of MT on cytokine profiles based on this review is to indicate that there is insufficient evidence to draw conclusions. In many studies investigating MT, results were mixed (29–31, 33, 56, 57, 59, 62, 82). The inconsistency across MT types, study designs, and dosages likely played a role in these mixed results. In addition, many of the subjects included were poorly described and it was unclear of the role of pathology on the biomarker response. Further study is warranted, but only if dosage rates are somewhat comparable to clinical applications assuring that they are at a level that might be therapeutically beneficial.

### Factors that identified variations in findings

We found that multiple factors seemed to influence response from exercise and MT (e.g., exercise intensity, duration, and an individual's overall health and fitness). The literature suggests that characteristics such as genetics, sex, age, and body composition interact with the immune system thereby influencing the inflammatory response (143–145). Intrinsic factors such as sleep quality (146–148), diet (149, 150), anxiety (151–153), depression (154, 155), fear (156), and stress (154, 157, 158) have all shown to associate with inflammatory mediator expression. Further, placebo/expectation-based effects have also shown to moderate inflammation in conditions including asthma, irritable bowel syndrome, chronic fatigue syndrome, and multiple sclerosis (159). Extrinsic factors have also been shown to influence neuroimmune responses including: auditory input (160), lighting (161), environmental pollution (162), time of day which measurement occurs (146, 163), and temperature (164). These confounding factors can demonstrate variability from day to day and therefore should be accounted for within mechanistic studies with measurement at baseline and re-assessment to establish association with mechanistic outcome.

### Challenges in measuring pro- and anti-inflammatory cytokines

Variability in mechanism-specific research on inflammatory cytokines stems from the complex interplay of the neurological, endocrine, and immune systems that regulate them. Population-based differences in immunologic profiles have also been established and should be accounted for when assessing variability in mechanistic trials. Differences have been demonstrated between healthy controls and individuals with pain therefore limiting the applicability of several of the included trials (155, 165). Further complicating interpretation is evidence that even in individuals experiencing pain, different inflammatory pain disorders demonstrate different cytokine profiles that mediate inflammation (likely associated with crosstalk) (166). *In-vitro* studies have been proposed to identify specific local mechanistic responses by removing crosstalk and limiting systemic influences; however, that approach limits real world applicability *in-vivo* studies (167–169). Animal studies allow for more control over extrinsic stimuli potentially impacting outcomes; however, differences between animal models and humans are likely (169, 170).

The immune system does not act in isolation but rather in conjunction with the nervous and endocrine systems (171, 172). These systems all demonstrate connectivity and responsiveness to physical, psychological, physiological and viral stress (173). This multisystem interface attempts to establish balance between inflammatory and anti-inflammatory mechanisms (171, 172). An example of this response occurs when inflammatory mediators are released in response to peripheral tissue injury sensitizing peripheral nerve endings, nociceptors release neuropeptides attempting to modulate various inflammatory mediators to protect and heal the injured tissue (163, 174).

Further complicating the interaction between these systems is the fact that cytokines can influence one another and replicate action at other sites, which has been termed “crosstalk” (167, 175). This phenomenon is not specific to one class of cytokine with influence reaching beyond inflammation and peripheral sensitization. Interleukin-6 type cytokines have shown to have crosstalk with IL-12, IL-35, IL-1, TNFα, glucocorticoids, glucagon, and insulin (159). Cytokines also have effects on Apolipoprotein E (glycosylated protein) influencing neurodegenerative and autoimmune disorders (176), dendritic cells (175), and leptin (177). The cytokines outlined within this review do not act specifically, but act systemically (178–180). When studying and accounting for crosstalk, it is crucial that researchers optimize assays, use multiple assays to avoid background signal and nonspecific interactions, and consider the use of bioinformatics tools and larger sample sizes to improve precision.

### Study limitations

By its nature, the narrative overview design allows for qualitative interpretations from authors who summarize the research which increases the risk for subjectivity. Nonetheless, our objectives were two-fold and included an assessment accounting for the variability observed. Although there are consistent trends in the role of acute and chronic exercise on pro- and anti-inflammatory cytokines, studies included animal models and human studies without health compromise. This is a limitation that is worth noting. Further, a lack of a comprehensive systematic selection process increases risk of missing key studies. Despite this, we include nearly 80 studies that investigated cytokines, including systematic reviews, which we feel represents an accurate overview of the literature.

### Recommendations for future research

Future studies investigating the role of MT or exercise on pro- and anti-inflammatory cytokines need to control for confounding variables and contextual influences to the whatever extent is possible. Pre-clinical efficacy studies may better control for these variables; however, being less reflective of clinical practice limits the applicability of such work. Clinical studies can be performed with careful controls for factors such as timing and contextual influences. Future studies should investigate subjects with specific MSK-disorders, since cytokine changes depend on the health/disease of the individual. Further, study of single cytokines is likely of less benefit, given the complicated constellation of their interplay and crosstalk occurs between cytokines. The use of cytokine response phenotypes is likely to provide greater value when determining the transferability of findings to clinical populations. Studies should attempt to correlate clinical value of mechanistic response to determine whether the changes that occur are of benefit.
